# Supplementary material for: Implementation fidelity of Ethiopia’s Malaria test-and-treat guideline amid a resurgence in Amhara Region: A mixed-methods study
Source: PLoS One. 2026 Apr 30;21(4):e0348088. doi: 10.1371/journal.pone.0348088 (PMC13132217; doi:10.1371/journal.pone.0348088)
Supplement: S1 Fig — Flow diagram showing the multi-stage sampling procedure used to select 53 health facilities (38 public and 15 private) from 19 woredas in Amhara Region, Ethiopia, in 2025. Of 236 woredas in the region, 166 had complete weekly malaria surveillance data and were screened for resurgence; 30 met the resurgence criterion, defined as a ≥ 50% increase in confirmed malaria cases in 2024 compared with 2022. Selected woredas were stratified by geographic zone, and facilities were sampled separately for the public and private sectors. (DOCX) [file pone.0348088.s001.docx]

| Amhara Region: 236 woredas |
| --- |

↓

| 166 woredas with complete weekly malaria surveillance data |
| --- |

↓

| Resurgence screening: ≥50% increase in confirmed malaria cases in 2024 versus 2022 |
| --- |

↓

| 30 woredas met the resurgence criterion |
| --- |

↓

| Stratified by geographic zone |
| --- |

↓

| 19 woredas selected by simple random sampling |
| --- |

↓

| Public health facilities   - 152 eligible facilities - 38 selected by simple random sampling | Private health facilities   - No complete regional registry - 15 selected by systematic sampling from the woreda health office lists |
| --- | --- |

↓

| Final sample: 53 health facilities 1 provider interviewed per facility |
| --- |
