# Supplementary material for: Implementation fidelity of Ethiopia’s Malaria test-and-treat guideline amid a resurgence in Amhara Region: A mixed-methods study
Source: PLoS One. 2026 Apr 30;21(4):e0348088. doi: 10.1371/journal.pone.0348088 (PMC13132217; doi:10.1371/journal.pone.0348088)
Supplement: S1 Table — Item-level specification of the composite fidelity score, including domain definitions, data sources, recall windows, scoring rules, and standardization procedures. (DOCX) [file pone.0348088.s002.docx]

| Construct/domain | Operational definition | Indicator/data source | Recall window | Scoring and standardization | Use in analysis |
| --- | --- | --- | --- | --- | --- |
| Composite implementation fidelity score | Overall facility-level implementation fidelity to the malaria test-and-treat guideline | Unweighted mean of standardized content, coverage, and frequency domain scores | Most recent routine reporting period and provider report at interview | Composite score = mean of 3 domain scores, each standardized to 0-100 | Primary outcome |
| Content (adherence) | Adherence to core diagnostic and treatment steps in the guideline | Derived from provider interview and targeted review of recent malaria register entries and/or prescriptions available during the visit | Current routine practice / most recent available records | Four binary items scored 0/1 and summed (range 0-4), then standardized: observed score / 4 × 100 | Included in composite fidelity score |
| Content item 1 | Suspected malaria cases are tested parasitologically before treatment | Provider report | Current routine practice | Yes = 1; No = 0 | Included in content score |
| Content item 2 | Species-specific treatment selection is consistent with the national guideline | Provider report cross-checked against recent register entries and/or prescriptions | Most recent cases available at visit | Evidence consistent with guideline = 1; not consistent/no evidence = 0 | Included in content score |
| Content item 3 | First dose of antimalarial treatment is observed at the facility when feasible | Provider report | Current routine practice | Yes = 1; No = 0 | Included in content score |
| Content item 4 | Key counseling messages are routinely provided (for example, treatment completion and return precautions) | Provider report | Current routine practice | Yes = 1; No = 0 | Included in content score |
| Coverage | Proportion of suspected malaria cases tested before treatment | Facility register review | Most recent routine reporting period available | Percentage already on 0-100 scale | Included in composite fidelity score |
| Frequency | Consistency of testing febrile/suspected malaria patients before treatment | One provider-reported Likert-type item | Preceding month | Response coded 1-5 and linearly transformed to 0-100 | Included in composite fidelity score; excluded in sensitivity analysis |
| Fidelity category | Facility-level fidelity classification | Composite score | — | High: ≥75; Medium: 50-74; Low: <50 | Descriptive analysis |
| Participant responsiveness | Provider acceptance of and active engagement with the guideline | Multi-item provider scale | Current routine practice | Items summed and standardized to 0-100; higher values indicate greater responsiveness | Independent variable |
| Facilitation strategies | Availability of implementation supports such as training, supervision, mentorship, job aids, and diagnostics/supplies | Multi-item provider/facility scale | Previous 3-24 months depending on item | Items summed and standardized to 0-100; higher values indicate stronger facilitation | Independent variable |
| Intervention complexity | Provider perception of how difficult the malaria test-and-treat strategy is to implement | One provider-reported item | Current perception | Original 5-point item reverse-coded so higher values indicate lower perceived complexity/easier implementation | Independent variable |
| Supply access | Availability of recommended diagnostics and antimalarial medicines | Facility report/observation | Previous 3 months | Binary or count-based indicator, as specified in the analysis dataset | Covariate |
| Supervision frequency | Number/frequency of supervision contacts related to malaria case management | Facility/provider report | Previous 6 months | Ordinal variable | Covariate / component of facilitation |
| Facility characteristics | Sector, facility level/type, guideline availability, diagnostic modality | Facility report/observation | At interview | Categorical indicators | Covariates |
| Provider characteristics | Sex, age, profession, years of service, malaria case-management experience | Provider interview | At interview | Categorical or continuous indicators | Covariates |
| Resurgence-related context | Stockout duration and district-level change in confirmed malaria cases | Facility report and surveillance data | Previous 3 months / 2022-2024 | Continuous indicators | Covariates |

Notes:

1. Standardization formula: observed score / maximum possible score × 100.
2. Equal weighting of content, coverage, and frequency was prespecified.
3. Sensitivity analyses excluded the self-reported frequency domain.
