# Supplementary material for: Implementation fidelity of Ethiopia’s Malaria test-and-treat guideline amid a resurgence in Amhara Region: A mixed-methods study
Source: PLoS One. 2026 Apr 30;21(4):e0348088. doi: 10.1371/journal.pone.0348088 (PMC13132217; doi:10.1371/journal.pone.0348088)
Supplement: S1 File — KoboToolbox questionnaire adapted from Carroll’s implementation fidelity framework and WHO malaria guidelines, covering provider and facility characteristics, fidelity domains (content, coverage, and frequency), and CFIR-aligned implementation moderators. The questionnaire was pilot tested in 3 facilities before data collection. (DOCX) [file pone.0348088.s003.docx]

Cover page fields

| Field | Entry |
| --- | --- |
| Zone | __________________ |
| Woreda | __________________ |
| Questionnaire ID | __________________ |
| Date of interview (dd/mm/yy) | __________________ |
| Facility name/code | __________________ |

Section A1. Provider and facility characteristics

| Item No. | Variable | Response options |
| --- | --- | --- |
| 1 | Facility sector | Public; Private |
| 2 | Respondent sex | Male; Female |
| 3 | Respondent age (completed years) | Numeric |
| 4 | Years of service/work experience (completed years) | Numeric |
| 5 | Years providing malaria diagnosis/treatment (completed years) | Numeric |
| 6 | Highest academic qualification | Certificate; Diploma; Degree; Second degree and above |
| 7 | Profession | Medical doctor; Pharmacist; Laboratory technician/technologist; BSc nurse; Clinical nurse; Other (specify) |
| 8 | Average number of malaria cases managed by the facility per month | Numeric |
| 9 | National malaria guideline available on site | Yes; No |
| 10 | Diagnostic modality available at the facility | None; Microscopy; RDT; Both microscopy and RDT |

Section A2. Provider knowledge of malaria

| Item No. | Question | Response options |
| --- | --- | --- |
| 11 | Can microscopic examination of a blood smear be used to diagnose malaria? | Yes; No |
| 12 | Are malaria rapid diagnostic tests (RDTs) reliable for detecting treatment failure within 21 days of a previous infection? | Yes; No |
| 13 | Are convulsions or loss of consciousness signs of uncomplicated malaria? | Yes; No |
| 14 | Should patients suspected of having uncomplicated malaria be tested to confirm the diagnosis? | Yes; No |
| 15 | Is Coartem (artemether-lumefantrine) the first-line drug for uncomplicated *P. falciparum* malaria? | Yes; No |
| 16 | Can artesunate be used to treat severe malaria? | Yes; No |
| 17 | Is primaquine given as a radical cure course for *Plasmodium falciparum* malaria? | Yes; No |
| 18 | Should healthcare workers refer severely ill malaria patients to higher-level facilities? | Yes; No |

Section B1. Implementation fidelity: content/adherence

| Item No. | Question | Response options |
| --- | --- | --- |
| 19 | Should parasitological diagnosis be performed for all suspected malaria cases? | Yes; No; Sometimes |
| 20 | What are the two recommended methods for parasitological diagnosis of malaria? (More than one response may apply.) | Microscopy and RDT; Clinical examination and RDT; Clinical examination and microscopy; Other (specify) |
| 21 | Should antimalarial treatment be initiated for patients whose diagnostic test result is negative? | Yes; No |
| 22 | Which of the following should a healthcare worker assess when managing a patient with suspected malaria? (More than one response may apply.) | Check for severity signs; Ask the patient’s age; Measure weight; Measure body temperature; Take additional history and perform physical examination as needed |
| 23 | Do healthcare providers in this facility strictly follow standard diagnostic procedures (for example, RDT or microscopy)? | Strongly disagree; Disagree; Agree; Strongly agree |
| 24 | Are treatment protocols for uncomplicated and severe malaria consistently applied according to the guideline in this facility? | Strongly disagree; Disagree; Agree; Strongly agree |
| 25 | Do healthcare workers demonstrate a clear practical understanding of malaria diagnosis and treatment? | Strongly disagree; Disagree; Agree; Strongly agree |
| 26 | Do you ensure that patients take the first dose of antimalarial medication at the facility? | Yes; No |
| 27 | What challenges, if any, do you face in adhering to the recommended diagnostic and treatment protocols? | Open-ended |

Section B2. Implementation fidelity: frequency

| Item No. | Question | Response options |
| --- | --- | --- |
| 28 | How often do healthcare workers conduct a malaria diagnostic test for suspected malaria cases before initiating treatment? | Never; Rarely; Sometimes; Often; Always |
| 29 | Does the facility organize refresher training on malaria diagnosis and treatment protocols? | Regularly (for example, quarterly or semi-annually); Occasionally (for example, annually or less frequently); Never |
| 30 | Is adherence to malaria treatment guidelines monitored during patient follow-up? | Always; Sometimes; Never |
| 31 | Is the availability of diagnostic tools and essential antimalarial medicines routinely assessed at the facility? | Yes; Sometimes; No |
| 32 | How often are healthcare workers trained or retrained on malaria diagnosis and treatment guidelines? | Never; Every 6 months; Once a year; Every 2 years |
| 33 | How often are quality-assurance activities conducted for malaria diagnostic tools (for example, RDTs or microscopy)? | Monthly; Quarterly; Annually; Never |

Section B3. Implementation fidelity: coverage

| Item No. | Question | Response options |
| --- | --- | --- |
| 34 | What percentage of suspected malaria cases undergo diagnostic testing before treatment in your facility? | Numeric percentage |
| 35 | To what extent are malaria diagnostic services accessible to all suspected cases in the catchment area? | 0-25%; 26-50%; 51-75%; 76-100% |
| 36 | What percentage of malaria treatment provided in your facility meets the national guideline? | Numeric percentage |
| 37 | What proportion of key target populations (for example, pregnant women and children aged <5 years) are adequately reached for malaria diagnosis and treatment? | Numeric percentage |

Section C1. Facilitation strategies

| Item No. | Question | Response options |
| --- | --- | --- |
| 38 | Have you received training in malaria case management and treatment in the previous 2 years? | Yes; No |
| 39 | Has the facility received malaria-related mentorship in the previous 3 months? | Yes; No |
| 40 | Are job aids, guidelines, or manuals available at the facility? | Yes; No |
| 41 | Has the facility been supervised on malaria case management and treatment in the previous 6 months? | Yes; No |
| 42 | If yes, how many supervision visits occurred in the previous 6 months? | One; Two; Three or more |
| 43 | Which recommended antimalarial medicines were in stock during the previous 3 months? | Artemether-lumefantrine; Injectable artesunate; Injectable quinine; Chloroquine tablets; Primaquine tablets |
| 44 | Were any non-standard antimalarial medicines in stock during the previous 3 months? If yes, please specify. | Open-ended |

Section C2. Quality of delivery

| Item No. | Question | Response options |
| --- | --- | --- |
| 45 | Do healthcare workers follow proper blood collection and handling procedures during malaria testing? | Yes; No |
| 46 | Do healthcare workers correctly interpret and document RDT or microscopy results for suspected malaria cases? | Yes; No |
| 47 | Are severely ill patients with malaria symptoms promptly referred to higher-level facilities? | Yes; No |
| 48 | Do patients receive counseling on completing antimalarial medication as prescribed? | Yes; No |

Section C3. Intervention complexity

| Item No. | Question | Response options |
| --- | --- | --- |
| 49 | How would you rate the complexity of the malaria test-and-treat strategy? | Very simple; Simple; Moderate; Complex; Very complex |
| 50 | What aspects of the strategy, if any, are challenging to implement in your setting? | Open-ended |
| 51 | Are there logistical challenges, such as supply chain problems or inadequate staffing, that affect implementation of the strategy? | Open-ended |

Section C4. Participant responsiveness

| Item No. | Question | Response options |
| --- | --- | --- |
| 52 | Do you differentiate malaria from other febrile illnesses that may present similarly? | Each time; Sometimes; Never |
| 53 | Do you ensure that prescribed treatment aligns with current malaria treatment guidelines? | Each time; Sometimes; Never |
| 54 | Do you educate patients on the importance of completing the full course of malaria treatment? | Each time; Sometimes; Never |
| 55 | Do you monitor for potential relapse or complications after initial malaria treatment? | Each time; Sometimes; Never |
| 56 | Do you maintain patient confidentiality while providing malaria care and treatment? | Each time; Sometimes; Never |
