# Supplementary material for: Implementation fidelity of Ethiopia’s Malaria test-and-treat guideline amid a resurgence in Amhara Region: A mixed-methods study
Source: PLoS One. 2026 Apr 30;21(4):e0348088. doi: 10.1371/journal.pone.0348088 (PMC13132217; doi:10.1371/journal.pone.0348088)
Supplement: S2 Table — Topic guide used for in-depth interviews, organized by CFIR-aligned domains. The guide was pretested with 2 providers, administered in Amharic, and translated into English as described in the Methods. (DOCX) [file pone.0348088.s004.docx]

| Question No. | Main question | Probes |
| --- | --- | --- |
| 1 | Can you describe how you follow malaria diagnosis and treatment guidelines in your daily practice? | What steps do you follow during diagnosis? How do you decide on the treatment regimen? Do you ever deviate from the guidelines? If yes, why? How do the guidelines influence adherence? Do the guidelines simplify clinical or referral decisions? |
| 2 | Do you believe that an experienced clinician can correctly diagnose malaria based only on clinical suspicion? | What factors affect a clinician’s ability to diagnose malaria by clinical suspicion? What are the advantages and disadvantages of diagnosing malaria without confirmatory tests? How often do experienced clinicians misdiagnose malaria, and why? |
| 3 | How do you involve patients in malaria diagnosis and treatment? | Do you explain the diagnosis and treatment plan to patients? How do you handle non-compliance or refusal of treatment? Are there strategies that improve patient adherence? |
| 4 | What quality-assurance measures are in place for malaria diagnosis and treatment? | Are diagnostic results regularly cross-checked, for example through microscopy quality assurance or supervisory review? How is treatment effectiveness monitored? Are there feedback mechanisms from supervisors or peers? |
| 5 | How do local factors influence implementation of malaria diagnosis and treatment? | Are there cultural or social factors affecting adherence? How do seasonal malaria trends affect your work? Does workload affect the quality of diagnosis and treatment? |
| 6 | Are the necessary resources for malaria diagnosis and treatment available in your facility? | Do you have sufficient diagnostic tools, such as RDTs or microscopes? Are recommended antimalarial medicines available? How do you handle shortages of tests or medicines? |
| 7 | What improvements would you suggest to strengthen implementation of the malaria test-and-treat strategy? | Probe for training, supervision, supplies, job aids, patient communication, private-sector support, and referral systems. |
| 8 | Is there anything else you would like to share about your experience with malaria diagnosis and treatment? | Open probe. |
| 9 | Please describe any external barriers to obtaining a confirmatory diagnosis of malaria. | Probe for availability of laboratory reagents and equipment, laboratory staffing, qualified personnel, electricity, workload, and referral barriers. |
