# Supplementary material for: Implementation fidelity of Ethiopia’s Malaria test-and-treat guideline amid a resurgence in Amhara Region: A mixed-methods study
Source: PLoS One. 2026 Apr 30;21(4):e0348088. doi: 10.1371/journal.pone.0348088 (PMC13132217; doi:10.1371/journal.pone.0348088)
Supplement: Attachment — Submitted filename: Response to Reviewers.docx [file pone.0348088.s006.docx]

**Response to the Academic Editor and Reviewers**

**Manuscript title:** Implementation Fidelity of Ethiopia's Malaria Test-and-Treat Guideline Amid a Resurgence in Amhara Region: A Mixed-Methods Study

**Manuscript ID:** PONE-D-25-41084

**Corresponding author:** Mastewal Worku

**Dear Editor and Reviewers,**

We thank the Academic Editor and Reviewer #1 for the careful review and constructive feedback. We revised the manuscript to (i) improve transparency and reproducibility of the implementation fidelity measurement, (ii) reduce risk of overfitting and clarify modeling decisions for the facility-level regression analysis, (iii) strengthen alignment of qualitative findings with CFIR constructs and integration with quantitative results, and (iv) ensure full compliance with PLOS ONE style, figure/map licensing, Supporting Information requirements, and data policy.

All changes have been incorporated into the revised manuscript and are addressed point-by-point below. Where possible, we indicate the location of revisions using section headings and page/line numbers.

**A) Academic Editor**

**1. PLOS style requirements**

**Comment:** Ensure the manuscript meets PLOS style requirements.

**Response:** We reformatted the manuscript to align with the PLOS ONE format, including the title page structure, heading levels, figure/table captions, and a dedicated Supporting Information section with captions and consistent labeling (S1 Table, S2 Table, S1 Fig).

**Manuscript location:** Title page; throughout manuscript; end of manuscript (Supporting Information section); all figure/table captions.

**2. Competing Interests wording**

**Comment:** Add the required sentence about adherence to PLOS policies.

**Response:** We updated the Competing Interests statement to: "The authors have declared that no competing interests exist. This does not alter our adherence to PLOS ONE policies on sharing data and materials."

**Manuscript location:** Competing Interests section.

**Also updated:** Cover letter and submission fields as applicable.

**3. Data availability restrictions**

**Comment:** Explain restrictions and provide the responsible contact body; confirm plan prior to acceptance.

**Response:** We adopted a restricted-access data-sharing approach. Although data are de-identified, the combination of facility-level indicators and qualitative narratives in a small, conflict-affected setting could enable deductive disclosure. In addition, unrestricted public deposition was not included in the consent process and IRB approval. We therefore expanded the Data Availability statement to (i) state the ethical rationale, (ii) name the restricting body (Bahir Dar University College of Medicine and Health Sciences Institutional Review Board, BDU-CMHS-IRB; Ref: BDU-CMHS-IRB 3053/2024), and (iii) provide a clear request process and contact details for data access.

**Manuscript location:** Data Availability statement.

**Note for the journal/submission system:** We have provided the IRB's official contact details ([cmhs@bdu.edu.et](mailto:cmhs@bdu.edu.et); telephone +251-58-899-9275) in the submission fields and can provide additional documentation upon request.

**Revised Data Availability statement:**

The data were collected by the authors and were not obtained from a third party. The facility-level quantitative dataset and qualitative interview data contain potentially identifying information. Because this study involved a small number of facilities in a conflict-affected setting, combinations of sector, facility type, district context, and stockout patterns, together with qualitative contextual details, may permit deductive disclosure even after removal of direct identifiers. Public sharing is therefore restricted by the Bahir Dar University College of Medicine and Health Sciences Institutional Review Board (BDU-CMHS-IRB; Ref: BDU-CMHS-IRB 3053/2024). Requests for the de-identified minimal quantitative dataset, codebook, and analysis code should be submitted to the BDU-CMHS-IRB, Bahir Dar University, Bahir Dar, Ethiopia, at [cmhs@bdu.edu.et](mailto:cmhs@bdu.edu.et) or telephone +251-58-899-9275. Requesters should provide the manuscript title, purpose of the request, planned analyses, and confirmation of appropriate ethics and data-security arrangements. Full qualitative transcripts cannot be shared publicly because they contain detailed contextual information that may permit identification; however, the qualitative codebook and selected de-identified excerpts are available from the same IRB upon approved request.

**4. Author name correction**

**Comment:** Add/correct author name "Kassahun Alemu Gelaye" in the submission data and manuscript.

**Response:** We corrected the author name to "Kassahun Alemu Gelaye" (previously listed as "Kasshun Alemu Gelaye") in both the manuscript author list and the submission metadata.

**Manuscript location:** Title page (author list).

**Submission system:** Author metadata updated.

**5. Figure 2 in-text citation**

**Comment:** Refer to Figure 2 in the text.

**Response:** We added an explicit in-text callout to Fig 2 in the Study Settings and Period section.

**Manuscript location:** Methods → Study Settings and Period.

**Revised sentence:** "Nineteen woredas were selected via simple random sampling, stratified by geographic zone to ensure regional representation (Fig 2)."

**6. Map copyright/licensing**

**Comment:** Ensure the map is CC BY–compliant and does not use copyrighted basemaps.

**Response:** We regenerated the map using openly available and CC BY–compatible administrative boundary layers from the Ethiopian Central Statistical Agency and Natural Earth. We revised the caption to include data sources and licensing/attribution consistent with PLOS ONE requirements.

**Manuscript location:** Fig 2 and caption.

**Revised caption:** "Fig 2. Map of Amhara Region showing the study woredas. The map was produced by the authors using QGIS software and publicly available administrative boundary layers from the Ethiopian Central Statistical Agency and Natural Earth (public domain). The map is original work and is provided under a CC BY 4.0 license."

**Also attached:** Separate map attribution and licensing documentation letter as requested.

**7. Table 7 in-text citation**

**Comment:** Cite Table 7 in the text.

**Response:** We added an in-text callout to Table 7 in the qualitative participant description section.

**Manuscript location:** Results → Qualitative study findings → Demographic characteristics (page 28, line 474).

**Revised sentence:** "In the full quantitative sample (N = 53), however, the pattern was reversed, with longer average service duration in the public sector (10.7 versus 7.5 years; see Table 7)."

**8. Supporting Information captions and citations**

**Comment:** Add Supporting Information captions at the end and ensure consistent in-text citations.

**Response:** We added a dedicated Supporting Information section at the end of the manuscript with captions for S1 Table, S2 Table, and S1 Fig, and aligned all in-text citations accordingly.

**Manuscript location:** End of manuscript (Supporting Information section); Methods (tool description) and Results (as cited).

**Supporting Information section:**

**S1 Table. Implementation fidelity measurement framework and operationalization.**
Detailed item-level specification of the composite fidelity score, including domain definitions, data sources (register-extracted, provider-reported), recall windows, scoring rules, and standardization methods. This table supports transparency and reproducibility of the primary outcome.

**S2 Table. Semi-structured interview guide for healthcare providers.**
Topic guide used for in-depth interviews, organized by CFIR-aligned domains, pre-tested before data collection. Administered in Amharic; translation protocol described in Methods.

**S1 Fig. Sampling flow diagram for the selection of woredas and health facilities.**
Flowchart illustrating the multi-stage sampling procedure used to select 53 health facilities (38 public, 15 private) from 19 woredas in Amhara Region, Ethiopia. Woredas were selected based on documented malaria resurgence (≥50% increase in confirmed cases, 2024 versus 2022) and stratified by geographic zone to ensure regional representation.

**9. Reference audit**

**Comment:** Ensure references are complete, non-duplicative, and correctly formatted; address any retractions if present.

**Response:** We audited the reference list, removed duplicates (including repeated entries for Carroll, Fetters, Rowe, Moran, Damschroder, Hasson, Bailey, and Gindola), replaced incomplete entries with complete WHO citations (including URLs where appropriate), corrected one mismatched reference (citation [3] now correctly cites WHO Guidelines for Malaria 2023), replaced one weak-fit US policy citation (former citation [63]) with an appropriate public-private health systems comparison study (Basu et al., 2012, PLoS Med), and standardized formatting throughout to PLOS ONE Vancouver style. We found no retracted references among those retained.

**B) Reviewer #1**

**Major Comment 1: Fidelity outcome construction and weighting**

**Comment:** The composite fidelity measure combines heterogeneous components; justify equal weighting; clarify item-level measurement; add sensitivity analyses excluding self-reported frequency.

**Response:** We expanded the Methods section to clearly define each fidelity domain (Content, Coverage, Frequency) and its component items. We added a detailed item-level specification table (S1 Table) that documents: data source (register/prescription review vs. provider self-report), recall window, scoring rules, and how domain-specific and overall composite scores were calculated.

We retained equal weighting as an a priori approach consistent with Carroll's implementation fidelity framework and the WHO test-and-treat strategy, which emphasize diagnostic access (Coverage), clinical adherence (Content), and sustained practice (Frequency) as equally critical domains. We acknowledge that this assumes equivalent contribution to implementation success.

We added and expanded a prespecified sensitivity analysis in which we excluded the self-reported Frequency domain and recalculated the composite fidelity score using only Content and Coverage. We repeated key comparisons (public vs. private) using this adjusted score. The direction and significance of results remained unchanged (p=0.038 vs. p=0.041), supporting the robustness of our findings.

We also expanded the Discussion to address social desirability bias, noting the vulnerability of self-reported frequency items and the mitigation steps taken (triangulation with register data, qualitative corroboration, and sensitivity analysis).

**Manuscript location:**

- Methods → Variables and measures → Primary outcome
- Methods → Variables and measures → Standardization and composite scoring
- S1 Table (Supporting Information)
- Results → Sensitivity analysis
- Discussion → Strengths and limitations

**New/added citations:**

- Carroll C, Patterson M, Wood S, Booth A, Rick J, Balain S. A conceptual framework for implementation fidelity. Implement Sci. 2007;2:40. <https://doi.org/10.1186/1748-5908-2-40>
- Hasson H. Systematic evaluation of implementation fidelity of complex interventions in health and social care. Implement Sci. 2010;5:67. <https://doi.org/10.1186/1748-5908-5-67>
- Krumpal I. Determinants of social desirability bias in sensitive surveys: a literature review. Qual Quant. 2013;47(4):2025-2047. <https://doi.org/10.1007/s11135-011-9640-9>

**Major Comment 2: Regression modeling (overfitting; resurgence covariates; bounded outcome)**

**Comment:** Risk of overfitting given n=53; define resurgence covariates; bounded outcome may violate OLS assumptions; clarify clustering/sampling.

**Response:** We clarified that the facility is the unit of analysis and that one provider was selected per facility to serve as the facility-level key informant, so clustering at the facility level is not applicable. We explicitly defined the resurgence-related covariates:
(i) facility-level stockout duration for first-line malaria diagnostics or antimalarial medicines during the preceding 3 months (continuous; provider-reported and cross-checked where stock records were available);
(ii) district-level percentage change in confirmed malaria cases between January–September 2022 and January–September 2024 (continuous; extracted from Amhara Public Health Institute surveillance summaries).

To reduce the risk of overfitting, we replaced stepwise backward elimination with a parsimonious, theory-driven hierarchical modeling approach. Model 1 includes facility sector, resurgence-related covariates, and basic facility/provider characteristics. Model 2 adds the three prespecified implementation moderators (participant responsiveness, facilitation strategies, intervention complexity). We report variance inflation factors (VIFs <2.5 for all predictors) to confirm the absence of problematic multicollinearity.

Because the fidelity score is bounded (0–100), we added diagnostic checks (residual plots, normality tests) and conducted a sensitivity analysis using fractional logistic regression (Papke & Wooldridge, 1996) after rescaling the outcome to (0,1). Results were substantively identical, so we present the linear model for interpretability while acknowledging the bounded nature of the outcome in the limitations.

**Manuscript location:**

- Methods → Participants and unit of analysis
- Methods → Variables and measures → Additional covariates
- Methods → Data analysis → Quantitative analysis
- Results → Factors associated with fidelity
- Discussion → Strengths and limitations

**New/added citations:**

- Papke LE, Wooldridge JM. Econometric methods for fractional response variables with an application to 401(k) plan participation rates. J Appl Econom. 1996;11(6):619-632. <https://doi.org/10.1002/(SICI)1099-1255(199611)11:6><619::AID-JAE418>3.0.CO;2-1
- Ferrari SLP, Cribari-Neto F. Beta regression for modelling rates and proportions. J Appl Stat. 2004;31(7):799-815. <https://doi.org/10.1080/0266476042000214501>

**Major Comment 3: Inconsistencies in medians, tests, and reporting**

**Comment:** Audit and correct inconsistent fidelity statistics; clarify composite vs domain-specific values; standardize test naming.

**Response:** We conducted a complete internal audit of descriptive and inferential outputs and corrected inconsistencies across the text, tables, and figures. We now report descriptive statistics (medians, IQRs) consistently in the narrative and in Table 4 (bivariate comparisons). Regression tables (Tables 5 and 6) are limited to model coefficients, confidence intervals, and p-values, with no redundant descriptive summaries.

We standardized test naming as "Wilcoxon rank-sum test (Mann–Whitney U)" throughout the manuscript and ensured that all reported test statistics, degrees of freedom, and p-values are consistent with the analysis outputs.

We also clarified that reported fidelity values refer to the composite score (0–100) unless explicitly labeled otherwise (e.g., "Content domain," "Coverage domain").

**Manuscript location:**

- Results → Fidelity to National Malaria "Test and Treat"
- Table 4
- Figure 5 caption
- Tables 5 and 6

**Major Comment 4: Qualitative CFIR framing and integration**

**Comment:** Qualitative results are descriptive; CFIR linkage is not explicit; strengthen mapping and integration with quantitative results.

**Response:** We clarified that we conducted an inductive thematic analysis to generate codes and themes from the data, followed by CFIR-informed mapping for interpretation. This approach allowed the analysis to remain data-driven while using CFIR to structure explanations of determinants.

We expanded Table 1 (CFIR Construct Mapping for Qualitative Coding) to explicitly link each theme and subtheme to specific CFIR domains and constructs (e.g., Inner Setting: Available Resources; Outer Setting: Patient Needs & Resources; Characteristics of Individuals: Knowledge & Beliefs).

We added illustrative quotations to strengthen the empirical grounding of each theme.

We strengthened the joint display (Table 8) to more directly connect qualitative themes (e.g., stockouts, diagnostic uncertainty, limited supervision) to the quantitative moderators (participant responsiveness, facilitation strategies, intervention complexity) and fidelity outcomes.

**Manuscript location:**

- Methods → Qualitative analysis
- Table 1: CFIR Construct Mapping for Qualitative Coding
- Results → Qualitative study findings
- Results → Integration of quantitative and qualitative findings

**New/added citations:**

- Damschroder LJ, Aron DC, Keith RE, Kirsh SR, Alexander JA, Lowery JC. Fostering implementation of health services research findings into practice: a consolidated framework for advancing implementation science. Implement Sci. 2009;4:50. <https://doi.org/10.1186/1748-5908-4-50>
- Fetters MD, Curry LA, Creswell JW. Achieving integration in mixed methods designs—principles and practices. Health Serv Res. 2013;48(6 Pt 2):2134-2156. <https://doi.org/10.1111/1475-6773.12117>

**Major Comment 5: Sampling transparency and documentation**

**Comment:** Clarify geographic units, how resurgence was operationalized, and the private sampling frame; add a flow diagram.

**Response:** We clarified the resurgence operational definition as a ≥50% increase in confirmed malaria cases in January–September 2024 compared with January–September 2022, based on weekly surveillance data from the Amhara Public Health Institute.

We described geographic units: 236 woredas (districts) in Amhara Region; 166 woredas with complete surveillance data were eligible for resurgence screening; 30 woredas met the resurgence criterion; 19 were selected via stratified random sampling across 8 geographic zones.

We clarified the sampling frames:

- **Public facilities:** selected via simple random sampling from a regional master list (152 eligible facilities identified; 38 sampled).
- **Private facilities:** selected using a two-stage approach because no complete regional private-facility registry was available: (1) woredas were randomly selected, and (2) private facilities within selected woredas were sampled systematically from district-maintained lists (15 sampled).

We added a sampling and participation flow diagram (S1 Fig) showing the progression from 236 woredas to the final sample of 53 facilities.

**Manuscript location:**

- Methods → Resurgence operational definition and sampling frame
- Methods → Participants and unit of analysis
- S1 Fig (Supporting Information; also cited in Methods)

**Minor comments: language, ethics detail, reliability, causal language**

**Comment:** Improve clarity, expand ethics procedures, report reliability where relevant, and avoid causal claims.

**Response:** We edited the manuscript for clarity and concision throughout.

We expanded the Ethics section to describe confidentiality procedures for interviews, including:

- Conducting interviews in private settings to maintain confidentiality
- Storing audio files on password-protected/encrypted devices
- De-identifying transcripts prior to analysis
- Deleting audio recordings after transcription
- Secure storage of signed consent forms

We reported scale composition and internal consistency (Cronbach's alpha) for multi-item moderator scales (participant responsiveness, facilitation strategies, intervention complexity) where applicable.

We revised wording throughout to avoid causal inference, using "associated with" rather than causal language (e.g., "predicted," "led to").

**Manuscript location:**

- Throughout manuscript (language edits)
- Methods → Ethical approval
- Methods → Data collection and analysis
- Discussion (revised interpretation to emphasize associations rather than causation)

**New/added citation:**

- Tavakol M, Dennick R. Making sense of Cronbach's alpha. Int J Med Educ. 2011;2:53-55. <https://doi.org/10.5116/ijme.4dfb.8dfd>

We appreciate the opportunity to revise and improve the manuscript. We believe these changes strengthen the transparency, methodological rigor, and policy relevance of the study while ensuring full compliance with PLOS ONE requirements. We would be glad to provide any additional information requested by the Academic Editor or Reviewers.

**Sincerely,**

**Mastewal Worku**, on behalf of all authors
